# Morphological and Immunohistochemical Characteristics of Liver Inflammation in Patients with a History of COVID-19

**DOI:** 10.3390/v18010068

**Published:** 2026-01-02

**Authors:** Ilze Strumfa, Ludmila Viksna, Oksana Kolesova, Ieva Vanaga, Haralds Plaudis, Jelena Storozenko, Boriss Strumfs, Janis Pavulans, Romans Uljanovs

**Affiliations:** 1 Department of Pathology, Riga Stradins University, 16 Dzirciema Street, LV-1007 Riga, Latvia; 2 Department of Infectology, Riga Stradins University, 16 Dzirciema Street, LV-1007 Riga, Latvia; 3 Institute of Microbiology and Virology, Riga Stradins University, 16 Dzirciema Street, LV-1007 Riga, Latvia; 4 Department of Surgery, Riga Stradins University, 16 Dzirciema Street, LV-1007 Riga, Latvia

**Keywords:** COVID-19, liver, hepatitis, regeneration, morphology

## Abstract

The COVID-19 pandemic caused more than seven million deaths, mostly via acute respiratory distress syndrome with microvascular thrombosis. Compared to the amount of information about pulmonary pathology, information about COVID-19-induced liver lesions is scarce, especially with regard to the long-term consequences. The aim of our study was to evaluate inflammatory, vascular and fibrotic changes in hepatobiliary tissues of patients with a history of COVID-19 (post-COVID-19 patients). Based on the Knodell score, moderate portal inflammation was observed in 41.2% of post-COVID-19 patients, contrasting with 14.3% of control cases (*p* = 0.06). Moderate periportal inflammation was present in 26.5% and 7.1% of patients, respectively (*p* = 0.08). Post-COVID-19 patients showed higher counts of CD3+ lymphocytes (*p* = 0.02) and lower counts of CD68+ macrophages (*p* = 0.04), as well as more frequent and extensive regenerative changes in hepatocytes and the biliary epithelium (*p* = 0.0007). We did not find significant fibrosis or pathological changes in blood vessels, and only mild steatosis was observed in both groups.

## 1. Introduction

The global outbreak of coronavirus disease 2019 (COVID-19), triggered by Severe Acute Respiratory Syndrome Coronavirus 2 (SARS-CoV-2), caused one of the deadliest pandemics in recent history, claiming more than seven million lives [1]. The virus targets angiotensin-converting enzyme 2 (ACE2) [2,3], which is highly expressed in the pulmonary alveolar epithelium (type 2), as well as in the endothelium [4]. Thus, acute respiratory distress syndrome with widespread pulmonary microvascular thrombosis is among the best-known manifestations of severe COVID-19 [5]. Given the frequent and severe lung damage that occurs in COVID-19 patients, most early COVID-19 studies focused on analysing pulmonary processes. However, biochemical signs of hepatocellular injury, e.g., elevated blood levels of alanine aminotransferase and aspartate aminotransferase, were often observed in the acute phase of COVID-19 [6,7,8]. The pathogenesis of these changes remained unclear, hypothetically including viral-mediated cytopathic injury, hypoxia-induced damage, immune-mediated injury, or side effects of intense treatment [6]. Despite controversies [9], expression of ACE2 has been described in hepatobiliary tissues [10]. In an animal model, viral replication in hepatocytes [11] has been experimentally demonstrated. The ongoing research on COVID-19 pathogenesis has highlighted the importance of the endothelial damage, resulting in the concept of immunothrombosis [12]. Morphological data on acute COVID-19-induced liver injury in humans have mostly been obtained from autopsies; they reflect various features, among which steatosis, necroapoptotic cell death, inflammation in the lobules, endothelium or portal fields, as well as thrombosis, endothelitis and fibrosis predominate [7]. In addition to controversies regarding liver damage in acute COVID-19, there is an even more marked shortage of data on human liver pathology caused by COVID-19 in the long term. Certain hepatic viral infections have high potential to transform into a chronic active disease, and SARS-CoV-2 is known to have the capacity to induce prolonged health deterioration [13]. Thus, it is mandatory to assess the long-term hepatobiliary sequelae of COVID-19.

The aim of our study was to evaluate inflammatory, vascular, and fibrotic changes in hepatobiliary tissues of patients with a history of COVID-19.

## 2. Materials and Methods

### 2.1. The General Characteristics and Ethical Principles of the Study

The study was performed as a prospective morphological and immunohistochemical evaluation of tissue samples, obtained from the liver and biliary system of the enrolled patients in regard to the history of COVID-19. The study was carried out in accordance with the Declaration of Helsinki and was approved by the Research Ethics Committee of Riga Stradins University, Riga, Latvia (protocol No 4/354/2022; issued on 29 June 2022). All participants signed an informed consent form prior to their participation in the study. The patient data were treated confidentially and anonymously.

### 2.2. Patient Recruitment Strategy, Inclusion and Exclusion Criteria

To ensure that tissue samples could be obtained, the study focused on patients who had to undergo a clinically indicated laparoscopic cholecystectomy because of gallstone disease. All operations were performed only according to clinical indications. The inclusion criteria comprised age (at least 18 years), clinical justification for the aforementioned surgical treatment, and lack of contraindications for surgery, as well as informed consent to participate in the study. Children and mentally disabled persons were not included in the study, considering their limited ability to provide informed consent.

Patients were excluded from the study if they had a clinical history of diseases that could induce inflammation in the liver tissues, or if such diagnoses were disclosed by laboratory investigation within the frames of the current study. The clinical exclusion criteria comprised a preceding known diagnosis of chronic viral or autoimmune inflammation in liver and bile ducts (chronic viral hepatitis B or C, autoimmune hepatitis, primary biliary cholangitis, primary sclerosing cholangitis); genetic disorders affecting the liver (haemochromatosis, Wilson’s disease); liver cirrhosis; any other autoimmune disease not listed before; or the presence or history of any malignant tumour and/or preceding liver surgery. Within the study, all patients underwent laboratory investigation in order to detect viral hepatitis (HCV, HBV, and HAV), HIV infection, autoimmune hepatitis, haemochromatosis, Wilson’s disease, and hepatocellular carcinoma. Any positive finding led to exclusion of the current study and transfer for appropriate treatment.

Upon the initial enrolment, the clinical history of COVID-19 was evaluated in order to recruit a certain number of patients with a history of COVID-19 (further designated post-COVID-19 patients) and controls lacking such history. Within the framework of the study, prior SARS-CoV-2 infection was verified by serological investigation.

### 2.3. Tissue Processing, Embedding, and Staining

During the operation, cholecystectomy was performed and a sample of liver tissues (1 cm^3^) was obtained at least 4 cm apart from the fossa of the gall bladder. All the removed tissues were immediately fixed in neutral-buffered formalin. At grossing, representative samples from the gall bladder were taken, including the cystic duct, corpus and fundus of gall bladder. The liver fragment was submitted in its entirety for histological investigation. The tissue samples underwent further routine processing, embedding in paraplast blocks, and microtomy. The histochemical panel comprised visualisation by haematoxylin–eosin staining [14], and, for the liver samples, periodic acid–Schiff (PAS) reaction [15], Perls’ stain to detect the presence of ferric (Fe^3+^) iron deposits in tissues [16], and Masson’s trichrome method to highlight collagen [17], while immunohistochemistry was focused on typing of inflammatory cells and identification of anti-SARS-CoV-2 nucleocapsid protein and endothelium.

For IHC, three-micrometre-thick sections were cut by an electronic rotary microtome Microm HM 360 on electrostatically charged glass slides (Histobond, Marienfeld, Germany) and subjected to deparaffinisation in xylene and graded alcohols (Sigma-Aldrich Merck, Darmstadt, Germany). Heat-induced antigen retrieval was performed in a microwave oven (3 × 5 min.) using basic TEG (pH 9.0) buffer (DAKO Agilent, Santa Clara, CA, USA). After blocking of endogenous peroxidase (Sigma-Aldrich), the sections were incubated with primary antibodies at room temperature. The clonality, species origin, species specificity, working dilution, and incubation time of the applied primary antibodies (all from Abcam, Cambridge, UK) are listed in Table 1. The bound primary antibodies were detected by the enzyme-conjugated polymeric visualisation system EnVision linked with horseradish peroxidase (DAKO Agilent). For colour development, 3,3′-diaminobenzidine (DAKO Agilent) was used as a chromogen, followed by counterstaining with Meyer’s haematoxylin. Positive and negative quality controls were performed and reacted appropriately.

### 2.4. Morphological Evaluation and Scoring

Inflammation in the liver, including periportal and bridging inflammation and necrosis; intralobular inflammation, degeneration and focal necrosis; portal inflammation and fibrosis, was evaluated semiquantitatively according to the Histology Activity Index (HAI) by the Knodell score [18,19,20]. In addition to the signs of fibrosis, defined by HAI (portal fibrosis, bridging fibrosis and cirrhosis), perisinusoidal arachnoid fibrosis and focal fibrosis were evaluated as binary categorical variables (present vs. absent). Considering the potentially low amount of collagen, especially in arachnoid fibrosis, and the need for accurate scoring of fibrosis, Masson’s trichrome-stained slides were used to detect and score all the listed fibrosis-associated parameters. The inflammation in the gall bladder was classified as acute vs. chronic and was classified by intensity as mild vs. moderate vs. intense. The inflammatory cells were counted in immunostained slides via computer-assisted morphometry in 40 high-power microscope fields (Nikon Eclipse Ci-L, Nikon Corporation, Tokyo, Japan) using 400× magnification (with an optical field area of 0.16 mm^3^). NIS-Elements D software, version 4.0 (Nikon Corporation) was used to count manually annotated cells, which were identified by a pathologist (IS).

Regarding liver cell morphology, steatosis was graded using a four-tiered scale (grade 0 —non-significant steatosis, fat vacuoles found in less than 5% hepatocytes; grade 1 —mild steatosis, 5–33% hepatocytes contain fat vacuoles; grade 2 —moderate steatosis, 33–66% hepatocytes affected by fat vacuoles; grade 3 —severe steatosis, more than 66% hepatocytes contain fat vacuoles) as proposed by Brunt et al., 1999 [21,22]. Mallory bodies were sought for and evaluated as a binary categorical variable (present vs. absent). Regenerative changes in hepatocytes and biliary epithelium were assessed by presence and extent as a categorical variable (absent vs. present in scattered cells/portal fields vs. nodular). Cholestasis was detected based on the presence of bile pigment in hepatocytes and/or bile plugs in bile capillaries or portal bile ducts and evaluated as a binary categorical variable (present vs. absent).

Endothelial swelling, microvascular thrombosis, and concentric and eccentric vascular fibrosis were assessed as binary categorical variables (present vs. absent), followed by location (liver sinusoids, portal and central venules, portal arterioles in hepatic tissues; small veins, small arteries, capillaries in the wall of the gall bladder and biliary ducts).

### 2.5. Statistical Analysis

Descriptive and analytical statistics were applied (GraphPad by Dotmatics, Boston, MA, USA). Prior to the statistical analysis, the assumption check of normality was performed using the Shapiro–Wilk test. The descriptive data were expressed as mean ± standard deviation (SD) or relative frequency with 95% confidence interval (CI). The Chi square test was used to assess the statistical significance of differences in categorical variables, and the Mann–Whitney U-test was applied to compare the mean values. Differences were considered statistically significant if the *p*-value was <0.05.

## 3. Results

### 3.1. Demographical and Clinical Characteristics of the Patients

During the study period, 62 patients were enrolled at the mean age of 55.8 ± standard deviation (SD) 16.4 years (range, 21–86; 95% confidence interval: 51.7–60.0). Among them, there were 47 females and 15 males (Table 2).

The groups of post-COVID-19 patients and control cases did not differ by age, gender, body mass index (BMI), or levels of indicator enzymes, such as aspartate aminotransferase (ASAT) and lactate dehydrogenase (LDH), which were assessed before surgery (Table 3). There were no differences in the proportion of patients with acute or chronic cholecystitis and reported comorbidities such as cardiovascular diseases and diabetes mellitus, type 2, representing the most frequent concomitant diseases (Table 3). In both groups, all persons had received COVID-19 vaccinations in 2021. All post-COVID-19 patients had had COVID-19 in the time period from 2021 to 2022, on average 12 ± 4.2 months before the cholecystectomy. In all cases, the disease was treated on an outpatient basis, corresponding to mild-to-moderate severity as defined by the World Health Organisation [23]. There were no patients with a history of ICU admission, any modality of ventilator support, and/or supplemental oxygen treatment.

### 3.2. Inflammatory Changes

**Figure 1:**
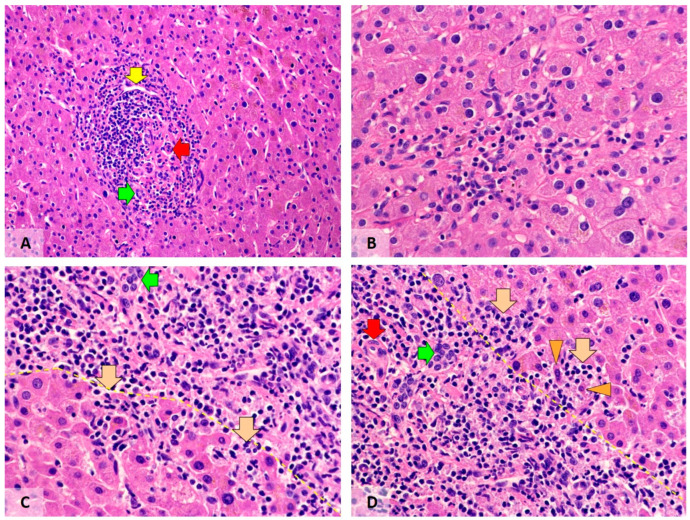
Inflammatory changes in liver tissues of post-COVID-19 patients. (**A**) Inflammation in a portal field. Green arrow, portal bile duct; red arrow, arteriole; yellow arrow, portal venule. (**B**) Lobular inflammation. (**C**,**D**) Different foci of periportal activity. Green arrows, portal bile duct; red arrow, arteriole; yellow dotted line, imaginary terminal plate line; brown arrows, periportal infiltrates; orange arrowheads, hepatocytes showing degenerative changes. Visualisation by haematoxylin–eosin stain; original magnification 100× (**A**) and 400× (**B**–**D**).

Both in post-COVID-19 patients and in the control group, the evaluated liver tissues showed mild-to-moderate polyclonal chronic inflammation with periportal activity in a fraction of patients, as reflected by the HAI according to the Knodell score (Table 4). Moderate (score 3 of 4) portal inflammation (Figure 1) was observed in 41.2% of post-COVID-19 patients, contrasting with 14.3% of control cases (*p* = 0.06). Moderate (score 3 of 4) periportal inflammation, affecting less than 50% of the portal field perimeter, was present in 26.5% and 7.1% of patients, respectively (*p* = 0.08).

Periportal, portal, and intralobular inflammatory infiltrates were composed of mononuclear cells. There were exceptionally rare neutrophils, eosinophils, and plasma cells in liver tissues. No granulomas or portal cholangitis were found. The cellular composition of portal and periportal inflammatory infiltrates is shown in Table 5.

### 3.3. Metabolic Changes: Steatosis and Cholestasis

**Figure 2:**
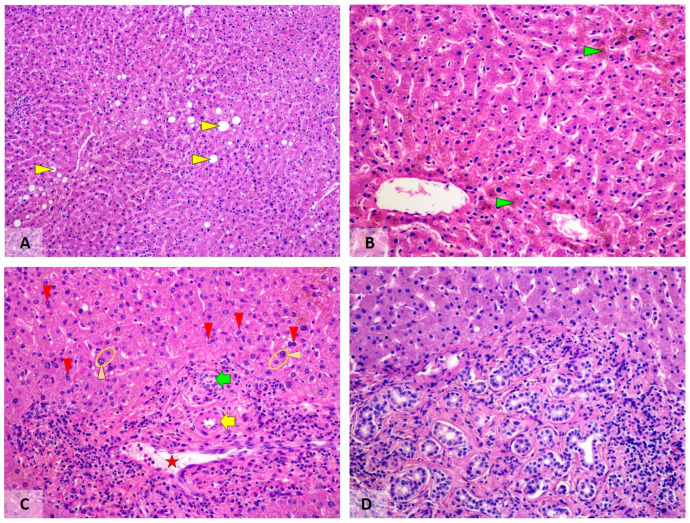
Metabolic and regenerative changes in liver tissues of post-COVID-19 patients. (**A**) Mild steatosis (yellow arrowheads). (**B**) Cholestasis (green arrowheads). (**C**) Regenerative changes in hepatocytes. Please note the binucleated liver cells (red arrowheads) and hepatocytes with enlarged nuclei (orange arrowheads). The green arrow highlights a portal bile duct; the yellow arrow points to an arteriole in the portal field; and the red star marks a portal venule. (**D**) Microhamartoma-like focus of bile duct proliferation in a portal field. Visualisation by haematoxylin–eosin stain; original magnification 50× (**A**) and 100× (**B**–**D**).

Liver steatosis (Figure 2) was observed in both groups (29.4% vs. 46.4%; *p* = 0.16), but invariably remained mild (<33%). Mallory hyaline and arachnoid fibrosis was not observed in any case. Morphological signs of cholestasis were evident in 23.5% of post-COVID-19 patients and 35.7% of controls; the difference lacked statistical significance with regard to a history of COVID-19 disease (*p* = 0.29) but showed an association with complicated gallstone disease (*p* = 0.04).

### 3.4. Morphological Manifestations of Hepatobiliary Regeneration

Signs of hepatocyte regeneration were statistically significantly (*p* = 0.0007) more frequent and extensive in post-COVID-19 patients. In six samples (17.7%), foci of regenerating hepatocytes and a single biliary microhamartoma-like focus were observed. The foci of regenerating hepatocytes were not associated with the presence of CD68+ or CD163+ macrophages or any other inflammatory infiltrate. Among the regenerative changes, abundant presence of binucleated hepatocytes, anisokaryosis, and ductular reaction were the most extensive and frequent findings, observed in 58.8%, 44.1, and 32.4% of post-COVID-19 patients, that statistically significantly (*p* = 0.001; 0.01 and 0.04, respectively) differed from occurrence in 25.0%, 14.3%, and 10.7% of control cases. Mitotic activity was rare, found only in two post-COVID-19 patients (5.9%).

### 3.5. Evaluation of Blood Vessel Morphology and Fibrosis

The liver sinusoids, portal arterioles, portal and central venules, as well as the arterioles of extrahepatic biliary ducts lacked morphological signs of microvascular thrombosis or organised mural or occlusive thrombi/thrombemboli (0/124 samples). Capillary thrombosis and endothelial swelling were limited to areas of acute inflammation caused by complicated gallstone disease. There was no evidence of immunohistochemical expression of SARS-CoV-2 nucleocapsid protein in endothelium or elsewhere.

There was no evidence of bridging fibrosis (fibrosis score 3 of 4 by hepatitis activity index (HAI) according to Knodell’s score) or liver cirrhosis (HAI fibrosis score 4). Focal fibrosis was observed in correlation with complicated gallstone disease (*p* = 0.02). Upon microscopic evaluation, the fibrotic foci lacked morphological colocalization with presence or groups of regenerative hepatocytes or more extensive ductular reaction.

## 4. Discussion

COVID-19, the disease caused by a recently discovered coronavirus SARS-CoV-2 infection, triggered a major worldwide outbreak that was classified by WHO as a Public Health Emergency of International Concern on 30 January 2020 and as a pandemic on 11 March 2020. As of 25 October 2025, WHO has reported 7’102’784 confirmed death cases, confirming the importance and danger of this infection [1]. The COVID-19 pandemic induced a broad spectrum of problems, ranging from intensive care of seriously ill patients to overload of medical systems, lockdowns, and psychosocial issues. The virus continues to circulate in the global community [1], although the mortality rate has significantly decreased. In addition, COVID-19 is a new disease and its pathogenetic mechanisms are not yet fully understood. A growing body of information indicates that COVID-19 can also induce prolonged changes in physiological functions, in the most severe cases manifesting as so-called long COVID—a syndrome that represents long-term health deterioration after acute SARS-CoV-2 infection. In 2024, the cumulative global incidence of long COVID was estimated to be as high as 400 million individuals [24]. Among the hypothetic pathogenetic mechanisms of long COVID, viral persistence, dysregulation of immune system, disturbances in mitochondrial function, abnormal activity of complement cascade, endothelial inflammation, and microbiome dysbiosis have been listed [24]. The still unclear long-term consequences of SARS-CoV-2 infection indicate the need for additional research. While neuropsychiatric [25,26,27,28], cardiovascular [29,30], and immune [31,32,33] systems, as well as their interface [34,35], have been widely evaluated for COVID-19-induced prolonged disturbances, less is known about liver lesions after acute COVID-19. However, certain hepatic viral infections have a high capacity for a chronic course [36] and the data on SARS-CoV-2 are insufficient in this aspect. Therefore, we planned our study, aiming to evaluate the morphological structure of liver tissues in patients with clinical and serological evidence of prior history of COVID-19.

To enter human cells, SARS-CoV-2 uses angiotensin-converting enzyme 2 (ACE2) as the receptor. ACE2 is highly expressed in the endothelium and type 2 alveolar epithelium, hence the predominance of pulmonary damage. In liver, expression of ACE2 is reported in endothelium of small blood vessels, but not sinusoids [37]. In early studies, Kupffer cells were found to lack ACE2 [37], and ACE2 levels in cholangiocytes were reported to exceed the expression in hepatocytes: 59.7% vs. 2.6%, respectively [38]. In a more recent study, ACE2 expression and productive replication of SARS-CoV-2 in human hepatocytes has been demonstrated [11]. Thus, hypothetically, COVID-19-induced cytopathic changes and inflammation could focus both on hepatocytes and portal bile ducts.

In autopsies, SARS-CoV-2 RNA has been identified in the liver [39,40,41]. In liver tissues of the deceased patients, congestion, portal lymphocytic infiltrate and steatosis [40,42,43,44] or shock-induced necrosis [45] have been noted in the background of severe pulmonary lesions, representing diffuse alveolar damage and widespread presence of fibrin microthrombi in capillaries [5,46]. In survivors of acute COVID-19, elevated levels of alanine aminotransferase ALAT and aspartate aminotransferase ASAT have been described [37], more frequently in severe, ICU-treated cases [47], while the reports on increased gamma glutamyl transferase GGT levels are limited [37]. Elevated bilirubin levels have been found as well [48]. A strong association between ASAT levels, severe course, and mortality has been identified [49]. Although the immunohistochemical evidence of ACE2 expression mostly highlights cholangiocytes as a possible target for COVID-19, biochemical blood tests indicate hepatocellular damage, reflected by increased concentrations of ALAT. Thus, the preceding evidence is partially contradictory with regard to the main target of liver inflammation in COVID-19 patients.

In our study exploring liver tissues of patients who had to undergo clinically justified cholecystectomy because of gallstone disease, we identified mild-to-moderate polyclonal chronic inflammation with periportal activity in a fraction of patients. Regarding the intensity and activity of inflammation, there were no statistically significant differences between post-COVID-19 patients and a control group lacking such history. However, there was a trend of more intense and active inflammation in the post-COVID-19 group. The presence of inflammation cannot be attributed to chronic viral hepatitis C or B, as these infections were serologically excluded by study criteria. The invariably mild degree of steatosis (in the absence of liver cirrhosis or even significant fibrosis) practically excludes non-alcoholic steatohepatitis (NASH) as the cause of inflammation [50]. Absence of Mallory hyaline, arachnoid fibrosis, and neutrophilic leukocytes virtually excludes alcohol-induced hepatitis [51]. There have been reports of autoimmune hepatitis or primary sclerosing cholangitis triggered by COVID-19 [8,52]. However, autoimmune liver diseases were excluded by study criteria, based both on medical history and serological data. The portal infiltrate showed only scant plasma cells that would not be characteristic for autoimmune hepatitis [53]. Morphologically, there was no evidence of ductopenia or florid lymphocytic cholangitis or granulomas in the portal fields, and thus a lack of support for a differential diagnosis of primary biliary cholangitis [54]. The lack of concentric periductal fibrosis, thickened basement membranes, and bile lakes does not support the hypothesis of primary sclerosing cholangitis [55]. Notably, the absence of classic serological and morphological findings still leaves the possibility of early or seronegative autoimmune disease, and such liver pathology has been reported in association with COVID-19. Thus, increased incidence of seronegative autoimmune hepatitis has been observed in children during the SARS-CoV-2 pandemic [56]. Seronegative autoimmune hepatitis has been reported after COVID-19 infection; the patient in question exhibited significant improvement after glucocorticoid treatment [57]. Hence, the exact cause of inflammatory changes in the liver remains unclear. Considering the presence of inflammation in both groups, the most likely explanation is inflammation associated with secondary changes induced by cholecystolithiasis. Hypothetically, post-COVID-19 patients might have had to delay their surgical treatment, resulting in more marked inflammation in liver tissues. In addition, SARS-CoV-2 can modulate the activity of the immune system [13], possibly delaying the clearance/supporting the persistence of inflammation. Further, our findings on the cellular composition of inflammatory infiltrate show statistically significant differences that are in line with immune modulation. Notably, we did not observe macrophages having foam cell morphology; this finding contrasts with studies performed on lung tissues in an animal model [58] and is likely to be associated with acute disease. In our experience, the portal and periportal inflammatory infiltrates in post-COVID-19 patients featured statistically significantly higher numbers of CD3-positive T lymphocytes, while the number of CD68-expressing macrophages was lower than in control cases. Further studies would be desirable for subtyping of immune cells.

Although microvascular thrombosis is an important component of severe acute COVID-19 [5] and endothelitis along with viral inclusions in endothelium have been identified in postmortem tissue samples [59], we did not find any evidence of current microvascular thrombosis or occlusive or mural microvascular fibrosis consistent with organised thrombi. Evidently, in survivors of COVID-19, minor thrombi might be subjected to thrombolysis. Alternatively, thrombosis in liver tissues could be limited due to lower levels of ACE2 in sinusoids [37]. Another significant “negative” finding is the mild degree of steatosis and absence of any trend towards more extensive steatosis in post-COVID-19 patients. Thus, the reported higher prevalence of steatosis in COVID-19 patients [8] may resolve over time or in association with regenerative changes in liver cells that were observed in our study. It should be noted that in our study, the frequency and grade of steatosis was low both in the post-COVID-19 patients and the control group. This finding is in accordance with relatively rare occurrence of comorbidity, such as cardiovascular pathology that might induce circulatory hypoxia, and type 2 diabetes mellitus. The frequency and spectrum of concomitant diseases is attributable to the study criteria, enrolling only patients who lacked contraindications for surgery.

Among the most marked differences between post-COVID-19 patients and the control group, there was significantly more frequent occurrence of regenerative changes in the post-COVID-19 patients, who occasionally demonstrated foci of regenerating liver cells and a single biliary microhamartoma. This is in agreement with previous reports that SARS-CoV-2 infection causes hepatocyte death [11] and in a mouse model, SARS-CoV-2 infects hepatocytes and cholangiocytes, inducing subsequent lymphocytic infiltration [11]. Interestingly, the pathogenesis of COVID-19-induced liver pathology has been attributed to multiple mechanisms, hypothetically including not only viral-mediated cytopathic injury, but also hypoxia-induced damage, immune-mediated injury, or side effects of intense treatment [6]. While all the explanations are reasonable, our post-COVID-19 patients invariably experienced a mild-to-moderate course of COVID-19: after the initial diagnostic evaluation (including routine detection of oxygen saturation in the blood), all of them were recommended to recover on outpatient basis. None of them underwent ICU treatment or had any kind of ventilatory support or oxygen supply, limiting the likelihood of hypoxia-induced damage or side effects of intense treatment.

Analysing the regenerative changes, abundant presence of binucleated hepatocytes, anisokaryosis, and ductular reaction were the most extensive and frequent findings, observed in 58.8%, 44.1, and 32.4% of post-COVID-19 patients; these findings statistically significantly (*p* = 0.001; 0.01 and 0.04, respectively) differed from occurrence in 25.0%, 14.3%, and 10.7% of control cases. Both binucleated hepatocytes and anisokaryosis are indicative of polyploidy, resulting from endomitosis: a non-canonical cell division in which cells enter mitosis but do not undergo cytokinesis [60]. Binucleation of hepatocytes can be associated with ageing [61]. However, in our study, there were no statistically significant age differences between post-COVID-19 patients and the control group; therefore, we linked this morphological finding with regeneration in accordance with Korchilava et al., 2025 [61,62]. Endomitotic formation of binucleated hepatocytes is associated with certain molecular mechanisms, including loss of membrane anchorage [60], dysfunction of insulin and p53/p21 signalling pathways [63], as well as alterations in Wnt molecular cascade [60] that is involved in the replication of SARS-CoV-2 [64], thus hypothetically linking binucleated morphology of hepatocytes with COVID-19.

The present study has certain limitations as well as features showing dual impact. Because of strict inclusion criteria and the surgery-based model, our study group is relatively small. The group size could influence the mathematical calculations, limiting the statistical significance to a trend and thus hindering the identification of true biological differences. In future research, it would be necessary to extend the study to a larger cohort. A modified scientific design could be considered in accordance with future hypotheses. Namely, if the active inflammation in liver tissues of our patients is attributable to SARS-CoV-2 infection, it would be more clearly demonstrable in a setting that excludes any secondary origin of liver inflammation, including gallstone disease. However, the presence of gallstone disease in our study model could be a strength, not a limitation, if COVID-19 acts as an immunomodulating factor. Further, all our post-COVID-19 patients had a history of mild-to-moderate disease, which was treated on an outpatient basis and could be associated with less intense liver damage. Severe COVID-19 would hypothetically induce more marked changes in hepatobiliary tissues, but would be associated with multiple bias, including the previously noted impact of hypoxia and side effects of administered treatment, as well as concomitant extrahepatic pathologies.

## 5. Conclusions

History of COVID-19 is not associated with statistically significantly more extensive or active inflammation in liver tissues or biliary pathways. However, there is a trend of higher periportal inflammatory activity and density of portal inflammation in the post-COVID-19 group that may have a biological significance. Along with statistically significant differences in the cellular composition of inflammatory infiltrate; absence of immunohistochemically detectable viral proteins; and history of mild-to-moderate COVID-19, which has been treated exclusively on an outpatient basis (avoiding ICU and oxygen supply), these findings indicate that COVID-19 has a long-term immunomodulatory role. The signs of hepatocyte regeneration and ductular reaction indicate active repair that is statistically significantly more marked in the post-COVID-19 group. In the current study, we did not observe significant long-term vascular or fibrotic pathological changes in the hepatobiliary tissues of post-COVID-19 patients.

## Figures and Tables

**Table 1:** The applied panel of primary antibodies for immunohistochemical visualisation: characteristics of the antibody and dilution.

Antigen	Antibody Characteristics	Clone	Dilution	Incubation, min.
CD3	MRAH	SP162	1:100	60
CD20	MRAH	SP32	1:100	60
CD31	MRAH	EPR17259	1:500	60
CD68	MMAH	KP1	1:2000	60
CD163	MRAH	EPR19518	1:500	60
SARS-CoV-2 NCP	MR	EPR24334-118	1:1000	60

Abbreviations in the Table: CD—cluster of differentiation; NCP—nucleocapsid protein; MRAH—monoclonal rabbit antibody against human antigen; MMAH—monoclonal mouse antibody against human antigen; MR—monoclonal rabbit antibody.

**Table 2:** Age and gender structure of the study participants.

Patient Category	Number	Age, Years		
		Mean ± Standard Deviation	Range	95% Confidence Interval
All	62	55.8 ± 16.4	21–86	51.7–60.0
Females	47	54.5 ± 16.6	21–86	49.6–59.4
Males	15	59.9 ± 15.6	34–81	51.3–68.6
Post-COVID-19 patients	34	55.4 ± 15.6	26–86	49.9–60.8
Controls	28	56.4 ± 17.6	21–83	49.6–63.2

**Table 3:** Clinical and demographic characteristics of post-COVID-19 patients and control group.

Parameters	Post-COVID-19 Patients	Control Group	*p* Value
Age, years (M ± SD)	55.4 ± 15.6	56.4 ± 17.6	0.806
Females, N (%)	27 (79.4%)	20 (71.4%)	0.465
BMI, kg/m^2^ (M ± SD)	29.1 ± 5.1	29.7 ± 5.5	0.691
ASAT, U/L (M ± SD)	42 ± 22	56 ± 46	0.115
LDH, U/L (M ± SD)	212 ± 111	223 ± 92	0.672
Acute cholecystitis, N (%)	18 (52.9%)	14 (50.0%)	0.818
Cardiovascular diseases, N (%)	4 (11.7%)	5 (17.9%)	0.498
Type 2 diabetes mellitus, N (%)	2 (5.9%)	1 (3.6%)	0.673

Abbreviations in the Table: M—mean value; SD—standard deviation; N—number; BMI—body mass index; ASAT—aspartate aminotransferase; LDH—lactate dehydrogenase.

**Table 4:** Morphological features of liver and biliary tissues related to a history of COVID-19.

Morphological Parameter	Post-COVID-19 Patients N, Proportion (%) [95% CI]	Control Group N, Proportion (%) [95% CI]	*p* Value
**HAI by Knodell score**			
Periportal and bridging necrosis			
0	18/34; 52.9 [35.1–70.2]	22/28; 78.6 [59.1–91.7]	0.08
1	7/34; 20.6 [8.7–37.9]	4/28; 14.3 [4.0–32.7]	
3	9/34; 26.5 [12.9–44.4]	2/28; 7.1 [0.9–23.5]	
4	0; 0 [0.0–10.3]	0; 0 [0.0–12.3]	
5	0; 0 [0.0–10.3]	0; 0 [0.0–12.3]	
6	0; 0 [0.0–10.3]	0; 0 [0.0–12.3]	
10	0; 0 [0.0–10.3]	0; 0 [0.0–12.3]	
Lobular inflammation			
0	6/34; 17.7 [6.8–34.5]	4/28; 14.3 [4.0–32.7]	0.39
1	19/34; 55.8 [37.9–72.8]	19/28; 67.8 [47.7–84.1]	
3	9/34; 26.5 [12.9–44.4]	5/28; 17.9 [6.1–36.9]	
4	0; 0 [0.0–10.3]	0; 0 [0.0–12.3]	
Portal inflammation			
0	3/34; 8.8 [1.9–23.7]	5/28; 17.9 [6.1–36.9]	0.06
1	17/34; 50.0 [32.4–67.6]	19/28; 67.8 [47.7–84.1]	
3	14/34; 41.2 [24.7–59.3]	4/28; 14.3 [4.0–32.7]	
4	0; 0 [0.0–10.3]	0; 0 [0.0–12.3]	
Fibrosis			
0	22/34; 64.7 [46.5–80.3]	23/28; 82.1 [63.1–93.9]	0.13
1	12/34; 35.3 [19.8–53.5]	5/28; 17.9 [6.1–36.9]	
3	0; 0 [0.0–10.3]	0; 0 [0.0–12.3]	
4	0; 0 [0.0–10.3]	0; 0 [0.0–12.3]	
**Steatosis**			
Absent or insignificant (<5%)	24/34; 70.6 [52.5–84.9]	15/28; 53.6 [33.9–72.5]	0.16
Mild (5–33% of hepatocytes)	10/34; 29.4 [15.1–47.6]	13/28; 46.4 [27.5–66.1]	
Moderate (33–66% of hepatocytes)	0; 0 [0.0–10.3]	0; 0 [0.0–12.3]	
Severe (more than 66% of hepatocytes)	0; 0 [0.0–10.3]	0; 0 [0.0–12.3]	
**Cholestasis**			
Absent	26/34; 76.5; [58.8–89.3]	18/28; 64.3 [44.1–81.4]	0.29
Present	8/34; 23.5 [10.8–41.2]	10/28; 35.7 [18.6–55.9]	
**Regenerative changes**			
Absent	8/34; 23.5 [10.8–41.2]	20/28; 71.4 [51.3–86.8]	0.0007
Present	20/34; 58.8 [40.7–75.4]	7/28; 25.0 [10.7–44.9]	
Nodular	6/34; 17.7 [6.8–34.5]	1/28; 3.6 [0.1–18.4]	
**Manifestations of regeneration**			
Ductular reaction	11/34; 32.4 [17.4–50.5]	3/28; 10.7 [2.3–28.2]	0.04
Abundant binucleated hepatocytes	20/34; 58.8 [40.7–75.4]	7/28; 25.0 [10.7–44.9]	0.001
Anisokaryosis of hepatocytes	15/34; 44.1 [27.9–62.1]	4/28; 14.3 [4.0–32.7]	0.01
Mitotic activity in hepatocytes	2/34; 5.9 [0.7–19.7]	0; 0 [0.0–12.3]	0.19
Increased thickness of trabeculae	10/34; 29.4 [15.1–47.6]	2/28; 7.1 [0.9–23.5]	0.02
**Pathology of extrahepatic biliary tree**			
Acute inflammation	19/34; 55.8 [37.9–72.8]	15/28; 53.6 [33.9–72.5]	0.79
Chronic inflammation	27/29 *; 93.1 [77.2–99.2]	22/24 *; 91,7 [73.0–99.0]	
Epithelial atrophy/hypertrophy	27/29 *; 93.1 [77.2–99.2]	24/24 *; 100 [85.8–100.0]	

* The parameter could not be evaluated in cases featuring severe acute inflammation due to gallstone disease. Abbreviations in the Table: N—number; CI—confidence interval; HAI—Histology Activity Index (HAI) by the Knodell score.

**Table 5:** The cellular components of portal and periportal inflammatory infiltrates.

	Mean Cell Count Per 10 HPF ± Standard Deviation		
Parameter	Post-COVID-19 Patients	Control Group	*p* Value
CD3	314 ± 57	283 ± 45	0.02
CD20	236 ± 49	241 ± 57	0.71
CD68	31 ± 19	42 ± 32	0.04
CD163	5 ± 14	4 ± 17	0.80

Abbreviations in the Table: CD—cluster of differentiation; HPF—high-power field (of microscope; magnification 400×).

## Data Availability

The original contributions presented in this study are included in the article. Further inquiries can be directed to the corresponding author.
